# Single-polyp metabolomics reveals biochemical structuring of the coral holobiont at multiple scales

**DOI:** 10.1038/s42003-023-05342-8

**Published:** 2023-09-26

**Authors:** Ty N. F. Roach, Shayle B. Matsuda, Christian Martin, Gintare Huckeba, Joel Huckeba, Valerie Kahkejian, Erika P. Santoro, Anneke van der Geer, Crawford Drury, Robert A. Quinn

**Affiliations:** 1 Hawaiʻi Institute of Marine Biology, University of Hawaiʻi at Mānoa, Kāneʻohe, HI USA; 2 Daniel P. Haerther Center for Conservation and Research, John G. Shedd Aquarium, Chicago, IL USA; 3 Department of Biochemistry and Molecular Biology, Michigan State University, East Lansing, MI USA; 4 Institute for Biodiversity and Ecosystem Dynamics (IBED), University of Amsterdam, Amsterdam, The Netherlands; 5 Red Sea Research Center, King Abdullah University of Science and Technology, Thuwal, Saudi Arabia; 6 Department of Biology, San Diego State University, San Diego, CA USA

**Keywords:** Molecular ecology, Metabolomics

## Abstract

All biology happens in space, and spatial structuring plays an important role in mediating biological processes at all scales from cells to ecosystems. However, the metabolomic structuring of the coral holobiont has yet to be fully explored. Here, we present a method to detect high-quality metabolomic data from individual coral polyps and apply this method to study the patterning of biochemicals across multiple spatial (~1 mm - ~100 m) and organizational scales (polyp to population). The data show a strong signature for individual coral colonies, a weaker signature of branches within colonies, and variation at the polyp level related to the polyps’ location along a branch. Mapping metabolites to either the coral or algal components of the holobiont reveals that polyp-level variation along the length of a branch was largely driven by molecules associated with the cnidarian host as opposed to the algal symbiont, predominantly putative sulfur-containing metabolites. This work yields insights on the spatial structuring of biochemicals in the coral holobiont, which is critical for design, analysis, and interpretation of studies on coral reef biochemistry.

## Abstract (web summary)

A method for obtaining high-quality metabolomics data from individual coral polyps is developed and subsequently applied to investigate biochemical structuring of the coral holobiont at multiple scales.

## Introduction

Spatial patterns in natural communities are an illustration of the processes that shape them. These patterns in biological systems emerge due to a combination of both biotic and abiotic factors. Just as advances in remote sensing allow for the exploration of increasingly larger scales, advances in molecular methods now facilitate the investigation of decreasingly smaller scales. Molecular variation at the atomic level can now be revealed from single cells to organisms^1^ to whole ecosystems^2^.

Despite a relatively small spatial footprint (~280,000 km^2^), coral reefs are one of the most diverse and productive ecosystems^3^. Processes such as dispersal, community interactions, and disturbances act together with environmental factors to create spatial signatures on the reef landscape^4^. At the macroscale, spatial dimensions vary from 10 s to 1000 s of meters, defining reef-wide patterns of organization^5^. At the mesoscale (i.e., meters to centimeters) corals often form their own local patterning^6–8^. The patterns seen on the macro and meso-scale in coral reefs are often a product of microscale structuring on a single coral colony^9^, where the coral holobiont creates micro-environments which host unique viral, microbial, and biochemical assemblies^2,7,10,11^. However, the extent to which coral biochemistry changes within and between scales has yet to be thoroughly addressed.

Here, we developed a metabolomics approach to investigate coral biochemistry starting from the fundamental organizational unit of a coral—the polyp. We analyzed the metabolomes of individual polyps from multiple branches across multiple colonies within a reef, to assess the spatial distribution of biochemicals across several spatial (~1 mm–100 m) and organizational (polyp, branch, colony, population) scales. Understanding the variability and spatial distribution of biochemicals across scales on coral reefs provides insight into the spatial ecology of the coral holobiont, which is critical for experimental design and data interpretation in future research.

**Fig. 1:**
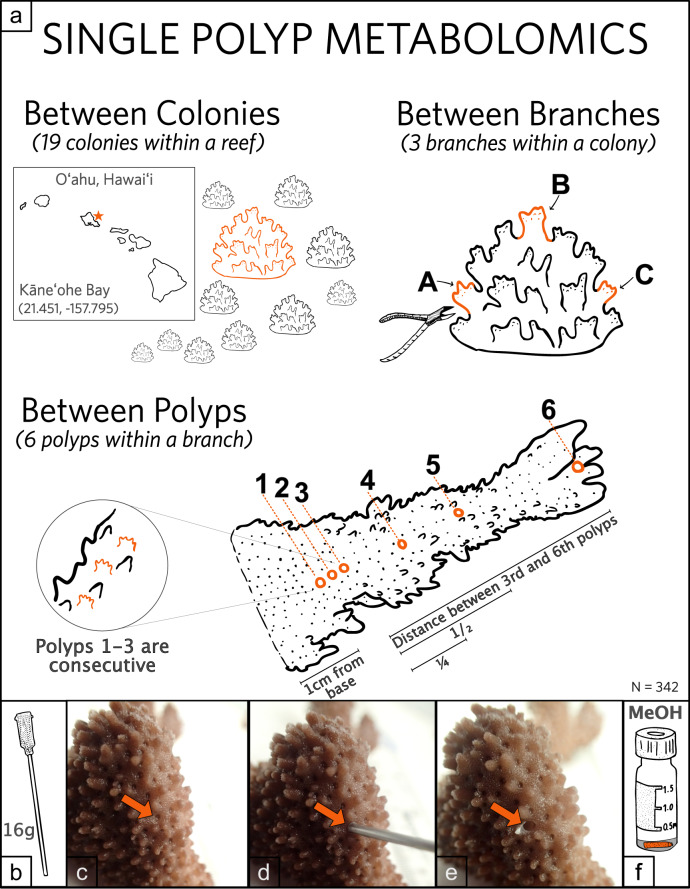
Single-polyp sampling scheme. **a** Sampling scheme where 19 *Montipora capitata* colonies were selected from Reef 13 (21.451, −157.795) in Kāneʻohe Bay, Oʻahu, Hawaiʻi. Three branches were clipped from each colony, and six single polyps were sampled from each branch for a total sample size of 342 polyps. **b**–**e** Each branch was sampled at a single-polyp resolution using a 16-gauge probe needle. **f** Each single-polyp sample was ejected from the 16-gauge needle directly into an amber, glass vial containing 100 µl of 70% methanol.

For this study, three branches were collected from each of 19 *Montipora capitata* colonies on a patch reef (21.451, −157.795) in Kāneʻohe Bay, Oʻahu, Hawaiʻi. Two branches were sampled from opposite sides of the colony, and one from the center (Fig. 1a). Six single-polyp biopsies were removed from each branch with a 16-gauge, blunt-tipped probe needle (Grainger) by sampling coral tissue directly surrounding an individual corallite to ensure the isolation of a single-polyp. The sample was then removed from the needle by pushing air through a syringe directly into a 1.5-ml, amber glass vial containing 100 μl of 70% methanol (Fig. 1b–f). The branch sampling scheme was as follows: *Polyp 1*: 1 cm above the base of branch; *Polyps 2* and *3*: the next consecutive polyps from polyp 1 toward the tip; *Polyp 4*: ¼ distance between Polyp 3 and tip of branch; *Polyp 5*: ½ distance between Polyp 3 and tip; *Polyp 6*: tip of branch). This yielded a total sample set of 342 individual polyps from 57 branches across 19 coral colonies (Fig. 1). These samples were randomized and assigned arbitrary labels prior to mass spectrometry analysis.

Samples were processed for untargeted metabolomics analysis via liquid chromatography-tandem mass spectrometry (LC-MS/MS) as previously described in Roach et al.^12^. Data files were converted to mzXML format for being processed with MZmine 2.53, the Global Natural Product Social Molecular Networking (GNPS) web-based platform, and SIRIUS^13–15^. These files were then compared to samples of bleached corals and symbiont isolates using molecular mapping^12^ to identify the putative source of metabolites. (i.e., metabolites from the coral host, algal symbiont, or shared). Additionally, raw data files were analyzed through Compound Discover for putative molecular annotation^16^. For detailed methodology of the workflow and analysis, please see the “Methods” section below.

## Results and discussion

### This single-polyp method produces a robust, high-quality metabolomics data signal

LC-MS/MS analysis of single-polyps collected with our approach produced a robust metabolome profile, similar to that of the more traditionally sampled, larger coral nubbins (Supplementary Figs. S1 and S2). The 342 individual polyps collected from 19 coral colonies produced metabolome data with a total of 555 unique metabolite features not found in blanks of which 67 (12.07%) had an MS/MS spectral match to a known compound in the GNPS database (details of GNPS library hits available in Supplementary Data 1). These were then manually inspected for good MS/MS alignment and curated to remove non-biological compounds resulting in 52 compound annotations that are at level two according to the Metabolomics Standards Initiative in Sumner et al.^15^ (Supplementary Data 1). We also searched this data against the mzCloud database and found 50 reliable annotations (level two annotations, above an alignment score of 90%, Supplementary Data 2). To further classify metabolite features, we used the molecular family classifier software, *CANOPUS*, to assign compounds to molecular classes and found that 75.2% of the MS/MS spectra detected could be assigned to the Class level of the *ClassyFire* molecular taxonomy^17^, which are considered level three according to Sumner et al.^15^. This demonstrated that while most of the MS/MS spectra in our coral metabolomes did not have direct hits to the GNPS libraries, they could be more readily assigned to classes of compounds.

### Variation in coral metabolomes across spatial and organizational scales

To assess the differences in general metabolomic profiles we compared the richness and Shannon Entropy of samples. There were no significant differences in richness (ANOVA *p* = 0.11) or entropy (ANOVA *p* = 0.84) by the different sampling areas on a branch (i.e., polyp number). There was, however, a significant difference in both richness (ANOVA *p* < 0.0001) and entropy (ANOVA *p* < 0.0001) between the different colonies. Within a single colony there were significant differences (ANOVA *p* < 0.05) in richness and entropy by branch in 8 out of 19 colonies (42.1%).

**Fig. 2:**
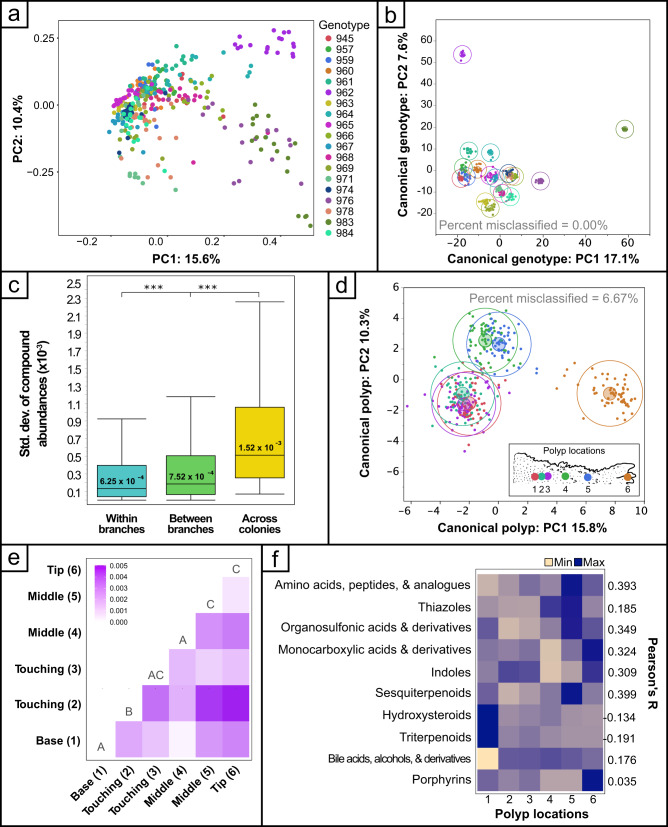
Single-polyp statistical analyses. **a** Principal component plot and (**b**) canonical plot from discriminant analysis supervised by colony using all metabolites. Color legend for genotypes is the same for (**a**, **b**). **c** Box plots showing the mean standard deviation of the abundance of every compound in all samples (“across colonies”), between branches within a colony (“between branches”), and between polyps within branches (“within branches”); ***p < 0.05. Boxplots are median with quartiles and whiskers extending 1.5 IQR beyond quartiles. **d** Canonical plot from discriminant analysis supervised by polyp number (i.e., position on the branch with lower numbers being closer to the base of the branch). **e** Pairwise Bray–Curtis dissimilarity matrix for polyps on the same branch. Shared letters represent no statistically significant differences between groups (pairwise Kruskal–Wallace; *α* = 0.95). **f** Heat map of ClassyFire SubClass chemical groups that were significantly correlated with the distance from the base of the branch with Pearson’s *r* values shown on the right of the map.

PERMANOVA analysis demonstrated significant effects of colony (*p* ≤ 0.001) and polyp location (*p* = 0.015). The data were visualized in a principal component plot (Fig. 2a) displaying a strong signature driven by colony. Discriminant analysis supervised by colony validated this signature (Fig. 2b) with 100% of the samples being classified correctly. Colonies 962 and 983 were notable outliers in both analyses compared to the other colonies which clustered more tightly. Average within-colony variance (7.52 × 10^−4^) was less than half the average between-colony variance (1.52 × 10^−3^; *p* < 0.05) (Fig. 1f). In addition to the colony signature, there was a distinction between individual branches within a colony, with the average variance within a branch being significantly less than the average variance between branches from the same colony (Fig. 2c). Independent discriminant analyses for each colony supervised by branch demonstrated a range of misclassification rates (Min = 0%; Max = 55.56%) with an average misclassification rate of 27.19% (Std. Dev. = 21.01%). Despite the higher average misclassification for branches within a colony, it is notable that there was 0% misclassification for 6 out of the 19 colonies (31.58%). This indicates a highly significant signature of branches within a colony for some colonies, while less so for others.

### Signature of polyp location within a single coral branch

In addition, we found a significant signature of the polyp location (PERMANOVA *p* = 0.015), which was strengthened when the colony was considered (PERMANOVA *p* = 0.001). A canonical plot generated via discriminant analysis supervised by polyp number (i.e., sampling location on a branch) revealed that the samples formed three distinct clusters (base, middle, and tip of the branches; Fig. 2d). This analysis was even able to discriminate between adjacent polyps with high rates of accuracy (93.33%) (Fig. 2d). Furthermore, adjacent polyps 1 and 2 were found to be significantly dissimilar to one another (Fig. 2e). To provide a general assessment of the type of molecules changing with distance from the base of a coral branch, the *ClassyFire* classifications of each metabolite were compared to the distance from the base of the coral branch using linear regression. Only two molecular SubClass families were significantly negatively correlated with distance to base after Bonferroni *p* value correction (triterpenoids and hydroxysteroids) while numerous SubClasses increased in relative abundance as sample distance from the base increased including sesquiterpenoids, amino acids and derivatives, organosulfonic acids and derivatives, carbamate esters, and others (Fig. 2f, Supplementary Figs. S3 and S4 and Supplementary Data 2).

**Fig. 3:**
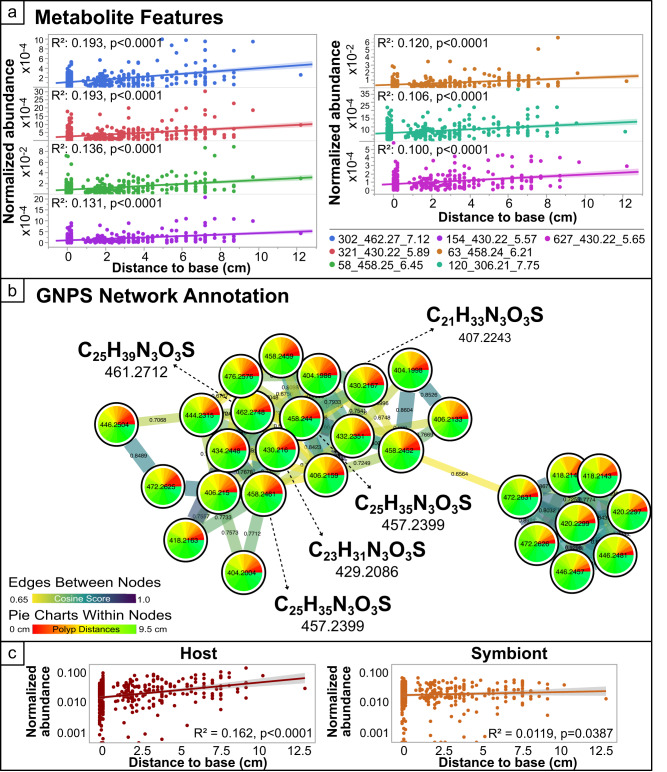
Metabolites significantly correlated with the distance from the base of a coral branch. **a** Linear regressions of all metabolites with a significant *R*^2^ > 0.10. Metabolite IDs shown in the figure panel represent the GNPS ID, followed by the mass charge ratio, and retention time. **b** Network and putative molecular formulas for the five highly correlated metabolites all in the same GNPS subnetwork. All masses listed are exact masses without adducts. A high-definition full page version of (**b**) is included in the Supplementary (Supplementary Fig. S6). **c** Linear regressions of the sum of all host-associated and algal-associated metabolites with the distance from the base of a coral branch.

Within branches, there was minimal correlation between pairwise sample dissimilarity (Bray–Curtis) and physical distance between samples (mean *R*^2^_adj_ = 0.0295), indicating little support for isolation by distance at this scale in the coral metabolome. However, a large portion of the variance among polyps within a branch was explained by the distance of the polyp from the base of the branch (artificial neural network regression analysis *R*^2^ = 0.83). Independent linear regressions of distance to the base with all biochemical features in the dataset were conducted, and seven biochemicals were significantly correlated (*p* < 0.05) with the distance to the base of the branch with an *R*^2^ > 0.10. Though none of these biochemical features had GNPS annotations, five of these seven compounds belonged to a single MS/MS network in GNPS (Fig. 3a, b). *SIRIUS*4.8.2^14^ was used to calculate in silico molecular formulas and structures for all five molecules of interest in the GNPS network. Many of the molecular formulas predicted were sulfur containing, thus, we analyzed the MS/MS spectra of one of the more abundant molecules (*m*/*z* = 458.2452, C_25_H_35_O_3_N_3_S) and were able to identify fragments in the low mass range that contained at least a single sulfur atom, providing further support for this molecular formula (Supplementary Fig. S5). Searching of the mzCloud library with Thermo® Compound Discoverer software (see “Methods”) also predicted the same molecular formula but had no annotation for the spectrum. Because the various cheminformatic approaches used to identify these molecules did not reveal plausible candidates, they remain structurally unknown (level-3 according to the metabolomics standards initiative^15^ and level 4 according to identification confidence levels^18^). MASST searching against GNPS public data^19^ with the MS/MS spectrum of the compound listed above revealed that this molecular spectrum (m/z458.2452) was found in four other datasets on GNPS, all coral-associated (see Supplementary Materials for link to these publicly available datasets), supporting its existence as a coral metabolite of interest.

### Metabolomic signatures within a branch are largely driven by host-derived molecules

To better describe the source of variation along the length of a branch we applied our holobiont metabolome mapping approach (originally developed in ref. ^12^) where LC-MS/MS data from bleached *M. capitata* and purified algal symbionts were co-networked on GNPS with this single-polyp data. MS/MS spectral matching across the two datasets enabled assignment of compounds as “host” if they were 10x more abundant in the bleached corals and as “symbiont” if they were at least 10x more abundant in algal pellets. Molecular mapping revealed all seven of the sulfur containing metabolites that correlated with branch length (Fig. 3a) were significantly enriched (Kruskal–Wallace *p* < 0.05) in the coral host relative to the algal symbionts. Furthermore, the sum of all host-associated metabolites was significantly correlated with the distance from the base of the branch (*p* < 0.001) with relatively strong predictive power (*R*^2^ = 0.159); whereas algal-associated metabolites were significantly less predictive of the distance along the branch with the *R*^2^ being an order of magnitude lower than for host-associated metabolites (*p* = 0.038, *R*^2^ = 0.009) (Fig. 3c).

### Further discussion and conclusion

This pattern may reflect the differentiation of growing apical polyps in Acroporidae or may be due to gradients in abiotic factors, such as light or flow. These findings provide important insight into the spatial variability and organization within and between coral polyps, branches, colonies, and populations. Understanding the amount of variability across micro- and macro-scales directly impacts our understanding of spatial structuring within and between scales of the coral holobiont^20,21^. As this work provide evidence for non-random structuring of the metabolome at multiple spatial and organizational levels, it offers valuable insight into the current debates concerning the variability and heterogeneity of metabolomes across scales, which is a critical component to consider when designing the approach to large scale ecological sampling schemes and interpreting data in future experiments.

## Methods

### Collection and sampling

Coral samples were collected in a single sampling event at patch reef #13 in Kāneʻohe Bay (21°30’49” N 157°55’03” W) on the windward side O’ahu, Hawai’i. Three branches, ~8–15 cm in length, were collected from each of 19 *Montipora capitata* colonies, at the same depth (~3 m), via SCUBA diving. Branches were chosen based upon their location within *M. capitata* colonies: two from opposite extremities, and one from the center. Each colony was photographed before and after sampling. Additionally, each sampled branch was photographed, assigned a unique field ID, and had its location mapped within its colony of origin. Samples were then stored in seawater at ambient oceanic temperature and returned to the Hawaii Institute of Marine Biology (HIMB) for processing, where they were held in flow through seawater tanks (all single-polyp samples were collected the same day as field collection in a single sampling event).

Six coral polyps were sampled from each coral branch for metabolomic analysis. Single-polyp biopsies were removed by taking a tissue punch with a 16-gauge blunt-tipped probe needle (Grainger) by sampling coral tissue directly surrounding the corallite to ensure the isolation of a polyp (Fig. 1 before/after tissue punch). The corallite was then removed from the needle by pushing air through a syringe directly into a 1.5 ml, amber glass vial containing 100 μl of 70% methanol solution. A blank sample was included using the same extraction solvent and sample vials but not coral sample. The samples were stored at −80 °C. The following describes the polyp locations within a branch starting with “Polyp 1”, 1 cm above the branch’s base. The next two samples (Polyps 2 and 3) were taken from consecutive polyps following the first up the branch (Fig. 1a, three polyps sampled in a row). The first three samples were taken to assess the metabolites associated with three coral polyps directly next to one another in a colony. The next three samples were chosen based upon the distance between polyp 3 and the tip of the branch (Fig. 1a, distance of 3rd polyp to branch tip). Polyp 4 was sampled ¼ of the total distance from Polyp 3 to branch tip, and Polyp 5 was ½ of the total distance from Polyp 3 to branch tip. Lastly, Polyp 6 was taken from the very end of the branch tip (Fig. 1a polyps 4–6 labeled on branch). The last three samples were taken to assess the effect of isolation by distance on polyp metabolites within a single coral branch. All samples were taken in a randomized fashion to help eliminate bias and sampling artifacts; furthermore, samples were randomized prior to mass spectrometry analysis and given arbitrary labels to blind them from the analyst.

### Mass spectrometry data collection and processing

The randomized methanol extracts were analyzed on a Thermo^TM^ QExactive^TM^ mass spectrometer coupled to a Vanquish Ultra High-Performance Liquid Chromatography (UHPLC) system. No other processing or purification of the extracted sample was performed except a centrifugation step for 30 s at 5000 × *g* was used to pellet debris. A volume of 25 μl of the methanol extract containing the single-polyp sample was added directly to a mass a 96-well sample plate and diluted 1:1 in 50% methanol containing an internal standard of 2.5 mg/ml phenol red (phenolsulfonphthalein). The mobile phase was 0.1% formic acid in Milli-Q water (channel A) and acetonitrile (channel B). The stationary phase was a reverse phase column Waters® Acquity® (Wood Dale, IL, USA) UPLC BEH C-18 column, 2.1 mm × 100 mm. The chromatographic runs were 12 min-long with linear gradients as follows: 0–1 min 2% B, 1–8 min 2–100% B. This 100% B solution was then held for 2 min followed by a switch to 2% B for the remaining 2 min. The injection volume was 10 μl, the flow rate 0.40 ml/min and the column temperature 60 °C. Full MS^1^ survey scans and MS^2^ mass spectra for five precursor ions per survey scan were collected using electrospray ionization in positive mode with a scan range set from *m/z* 100 to 1500 for the full MS mode (minutes 1–10 of run). Quality control standards were also prepared from a random pool of 10 samples of the single-polyp data. This QC mix was injected after every 12 samples of the MS run to monitor quality of the MS peaks and instrument performance. The mix was monitored throughout the run and a retention time drift from the first to last quality control standard peak was less than 0.01 min. Furthermore, an extraction blank containing our methanol extract, but no coral sample was included at the time of sampling and through the entire MS procedure to monitor background signals in our reagent and instruments. These signals were removed from the resulting metabolome feature table based on their presence in blank samples compared to coral samples. A molecule had to be on average 3x higher in the sample than blank to be maintained in the data.

Raw files (.raw) were converted to .mzXML format for analysis. All files were processed with MZmine 2.53 software, the Global Natural Product Social web-based platform (GNPS) and SIRIUS^13,14^. MZMine 2.53 parameters were set up as follows: feature extraction for MS^1^ and MS^2^ was performed for a centroid mass detector with a signal threshold of 5.0 × 10^4^ counts. Chromatogram builder was run considering a minimum height of 1.0 × 10^5^ and a *m*/*z* tolerance of 7 ppm. Chromatograms were deconvoluted with a peak duration range of 0.0 to 3.00 min and a baseline cut-off algorithm of 1.0 × 10^5^. Isotopic peaks were grouped with a *m/z* tolerance of 0.02 Da and a retention time percentage of 0.05. Detected peaks were aligned through Join Aligner Module considering 0.02 Da and retention time tolerance of 0.02 min. The resulting peak list was gap filled considering an intensity tolerance value of 0.001 ppm, 0.02 Da and retention time tolerance of 0.02 min. The data was converted to Mascot graphical format (.mgf) and a feature quantification table was generated for running feature-based molecular networking (FBMN) workflow on GNPS (Felix Nothias et al.^22^; Martin et al.^23^; Wang et al.^24^). This feature table included data from blank samples that were collected at the time of coral sampling using the same reagents and extraction solvents but did not contain a coral sample. Any metabolites detected in these blank samples had to be on average 3-times more abundant that those in blanks to be included in the feature table. After blank removal the abundance of each feature was normalized to the total feature abundances creating relative abundances for the metabolome data. FBMN was performed with a parent and fragment mass ion tolerance of 0.02 Da, a cosine score of 0.65 and a minimum matched peaks minimum of 4. Feature-based molecular networking job is available at: https://gnps.ucsd.edu/ProteoSAFe/status.jsp?task=3f5258d734374246a452591f23763b9f and raw files are available in MASSIVE (massive.ucsd.edu) as MSV000090806. The in silico molecular classification in Sirius (Dührkop et al.^14^) was performed for Orbitrap instruments considering isotope scores and a mass deviation of 5 ppm. Molecular formulas were searched within biological databases only as well as the compound structural identification. CANOPUS was also applied for determining predicted compound class of relevant molecular features^25^. All molecules that were classified were compared across the dataset after summing the total abundances of each molecule at the *Class* level or *most specific Class* level according to the ClassyFire molecular classification scheme^17^.

Additionally, raw files were processed through Compound Discoverer 3.3(Thermo^TM^) which generates a workflow tree that only allows connection with logical association for putative molecular annotation. Here, we applied retention time alignment, unknown peak detection and ion association, detection of background components unrelated to experimental samples, prediction of elemental composition, ChemSpider database searching, and mzCloud spectral library matching as described in Supplementary Table S1.

### Statistics and reproducibility

The dataset consisted of LC MS/MS data from 19 replicate coral colonies where three branches were chosen from each colony and 6 single-polyp samples were taken from each branch. This resulted in a total of 342 samples where each sample was considered a biological replicate.

Discriminant analysis, principal component analysis, linear regressions, and comparison of means and standard deviations were performed in JMP14 or R statistical software. All analyses were run using default settings except discriminant analyses, which were run using JMP’s preset “wide-linear, many column” setting. R package vegan was used to run PERMANOVAs (adonis2) and create PCOAs (Bray–Curtis distances). Linear mixed effects models were run using the R package lme4. Correlations coefficients between the CANOPUS ClassyFire molecular families and distance to base were calculated using Pearson’s *r* value and *p* values adjusted for multiple comparisons according to the Bonferroni method.

### Reporting summary

Further information on research design is available in the Nature Portfolio Reporting Summary linked to this article.

### Supplementary information

Supplementary Material

Description of Supplementary Materials

Reporting Summary

## Data Availability

All data for this project can be found at the following GNPS link: https://gnps.ucsd.edu/ProteoSAFe/status.jsp?task=3f5258d734374246a452591f23763b9f and the bleach mapping data are found at this GNPS link: https://gnps.ucsd.edu/ProteoSAFe/status.jsp?task=c6354b24afaf498c80a562cbd02a4818. Library hits spectral alignments are available at: https://gnps.ucsd.edu/ProteoSAFe/result.jsp?task=3f5258d734374246a452591f23763b9f&view=view_all_annotations_DB. The data and EMBO ontology compatible metadata are publicly available at the GNPS MassIVE server at MassIVE ID: MSV000090806. The standard deviations of each compound across organizational scales (i.e., branch, colony, population) used to make Fig. 2c are included as Supplementary Data 3.
