# Supplementary material for: Single-polyp metabolomics reveals biochemical structuring of the coral holobiont at multiple scales
Source: Commun Biol. 2023 Sep 26;6:984. doi: 10.1038/s42003-023-05342-8 (PMC10522574; doi:10.1038/s42003-023-05342-8)
Supplement: Supplementary file 1 — Supplementary Material [file 42003_2023_5342_MOESM1_ESM.pdf]

Supplementary Material:

Link to MASST Search Results:

https://gnps.ucsd.edu/ProteoSAFe/status.jsp?task=320c80f4e35345a884eec0cced27353b

Supplementary Figures.

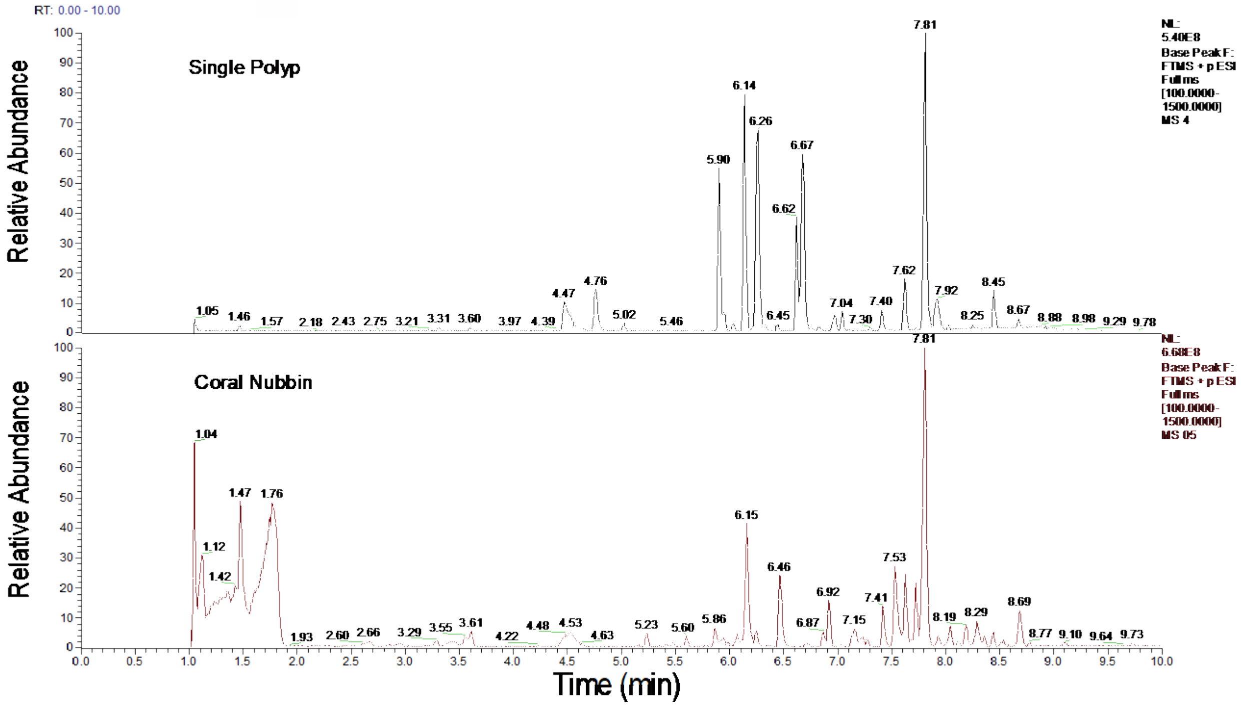

Figure S1. LC-MS total ion chromatogram of a single polyp sample collected from this study compared to a coral nubbin collected from Roach et al. 2021. The figure shows the robust metabolome signature obtained by our single polyp sampling approach.

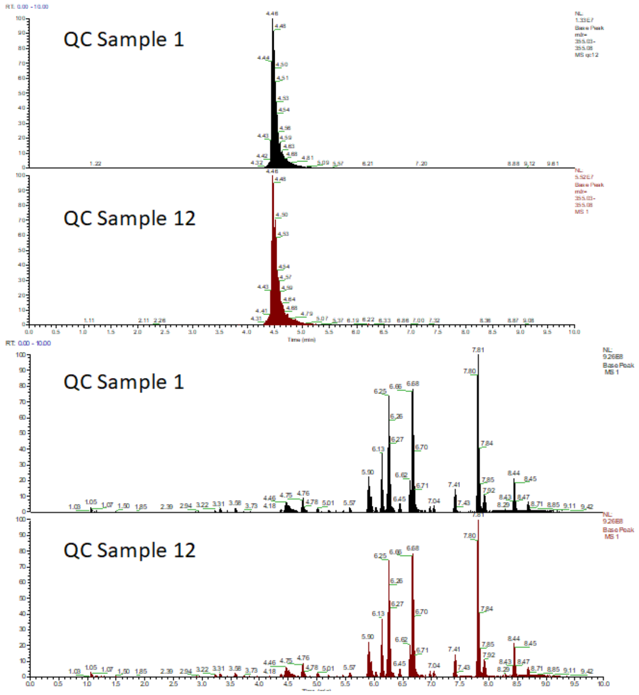

Figure S2. Total ion chromatogram of the phenol red internal standard and total ion chromatograms of the first and last Quality Control (QC) samples from the LC-MS/MS run.

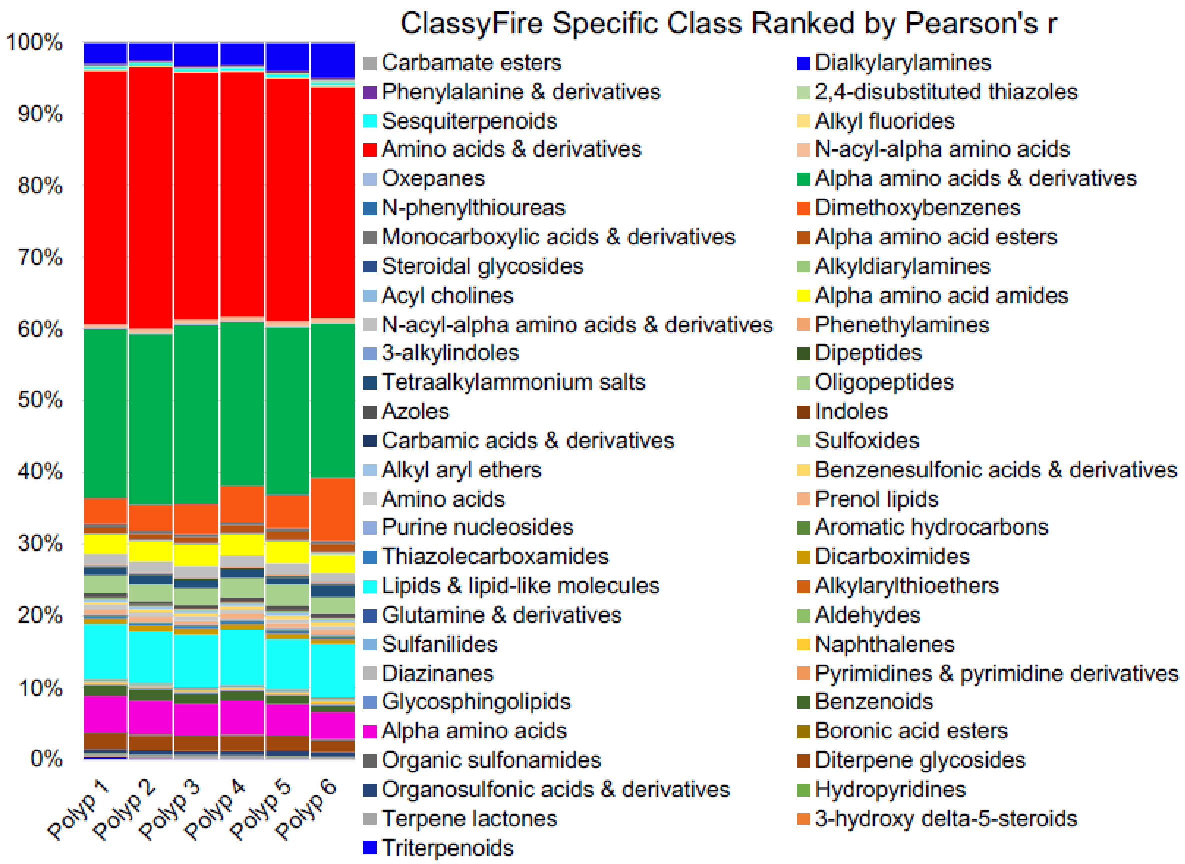

Figure S3. Stacked bar plot of ClassyFire most specific class chemical families that were significantly changing with distance from the base.

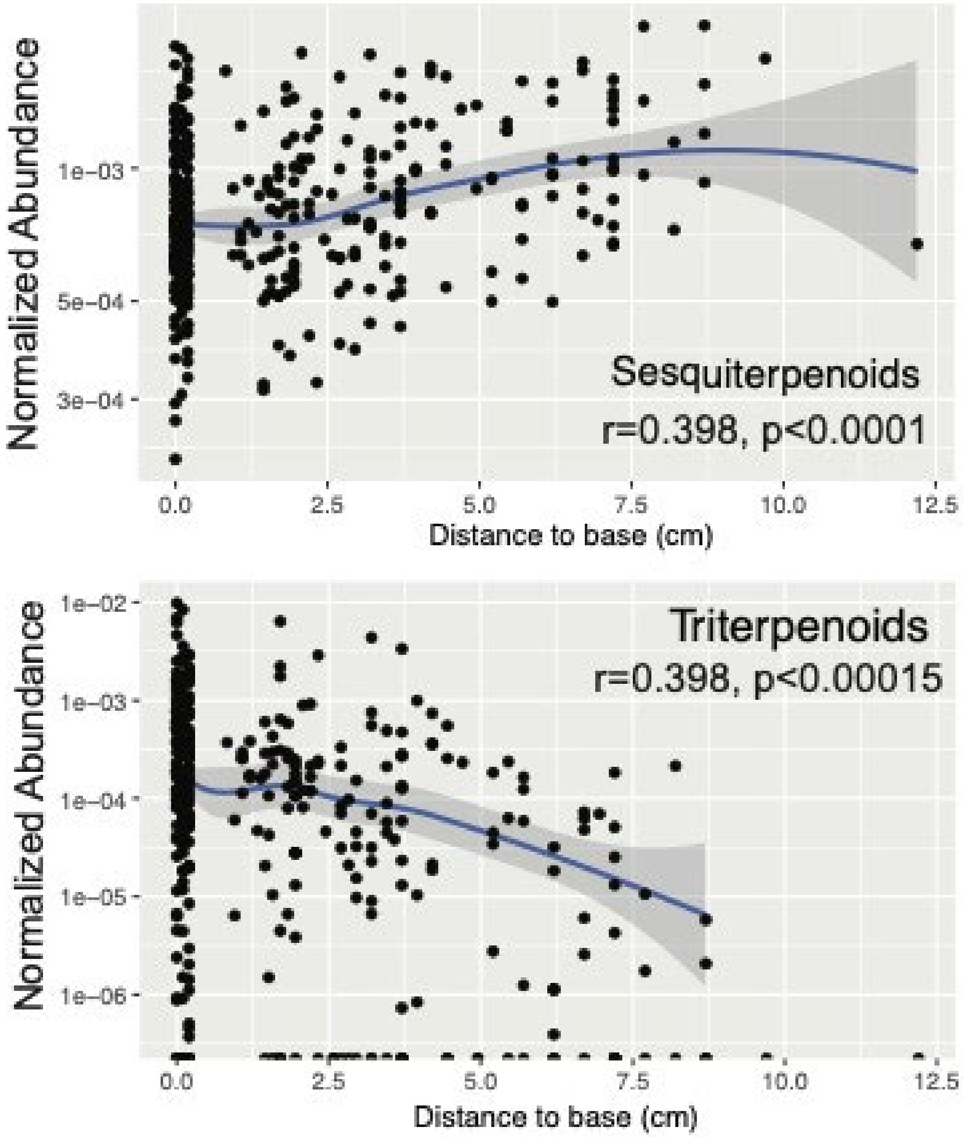

Figure S4. Normalized abundance of the total summed sesquiterpenoids and triterpenoids from CANOPUS output and distance from base-to-tip of a coral branch.

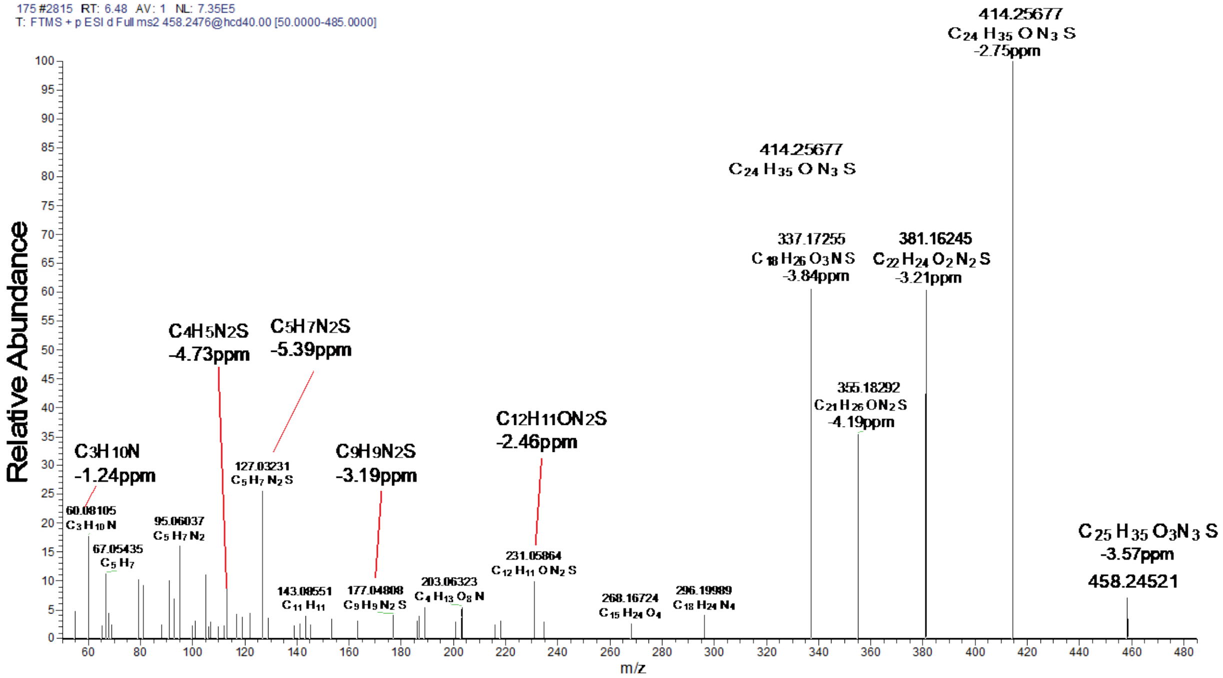

Figure S5. MS/MS fragmentation pattern of unknown sulfur containing compound associated with an increasing abundance from base to tip of coral branch. Molecular formula assignments and ppm errors of each fragment are shown to support the sulfur containing molecular formula of the parent ion.

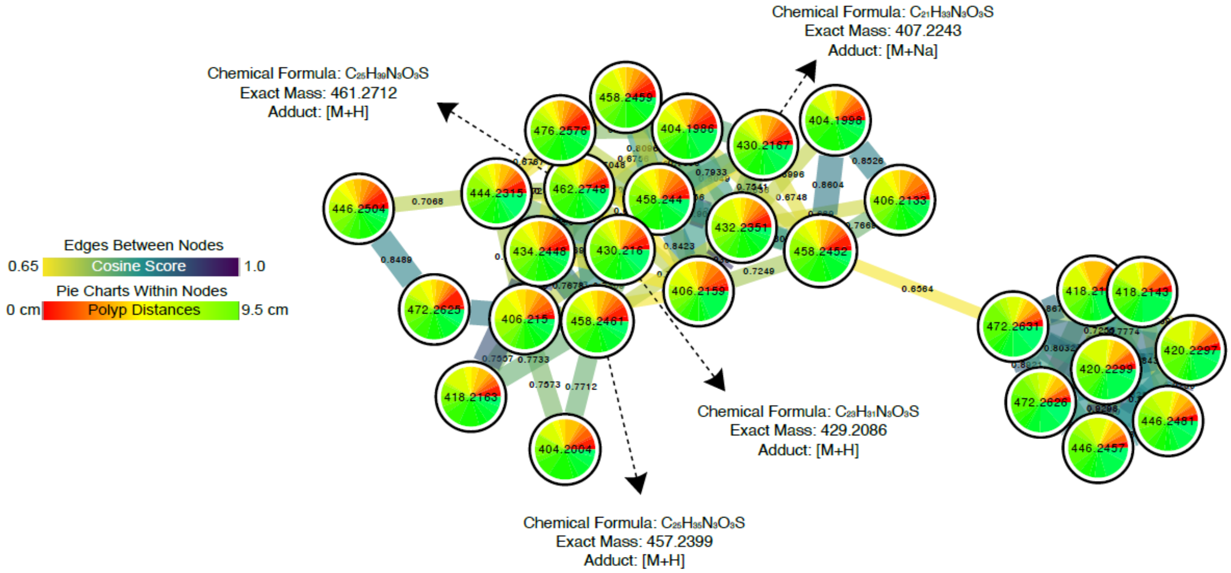

Figure S6. Network and putative molecular formulas for the five highly correlated metabolites all in the same GNPS subnetwork

Supplementary Tables.

Table S1. Compound discoverer (Thermo™) parameter applied for putative molecular annotation through ChemSpider database searching, and mzCloud spectral library matching.

Compound Discoverer™ 3.3 workflow	
Node	*Parameter*
Select Spectra	Lower RT limit = 1.0<br>Upper RT limit = 10.0<br>Polarity = Positive
Align Retention Time	Alignment model = Adaptative curve<br>Maximum shift = 0.2 min<br>Mass tolerance = 5 ppm
Detect Compounds	Mass tolerance = 5 ppm<br>Minimum peak intensity = $1.0e^{04}$<br>Most intense isotope = True<br>Chromatographic threshold = 1.5<br>Compound detection (ions) = [M+H] + 1; [M+K] + 1; [M+Na] +1; [M-H] -1
Group Compounds	Mass tolerance = 5 ppm<br>RT tolerance = 0.2 min<br>Prefered ions = [M+H] +1; [M-H] -1<br>Area of integration = Most common ion
Predict Composition	Mass tolerance = 5 ppm<br>Minimum element counts = CH<br>Maximum element count = C90H190Br3Cl4N10O18P3S5<br>Intensity tolerance for pattern matching = 30%<br>Intensity threshold = 0.1%<br>S/N threshold = 3<br>Use dynamic recalibration = True<br>Fragment matching = True

ChemSpider Searching	Database = KEGG<br>Search mode = Formula or Mass<br>Mass tolerance = 5 ppm<br>Maximum results per compound. = 100<br>Maximum predicted composition to be searched by compound = 3
mzCloud Searching	Compound classes = All<br>Library = Auto-processed; Reference<br>DDA library search = HighChem HighRes<br>Match activation type = True<br>Match activation energy = March with tolerance<br>Match activation tolerance 20<br>Apply intensity threshold = True<br>Match factor threshold = 60<br>Maximum isolation width (Da) = 500<br>Activation energy tolerance = 100<br>Match factor threshold = 20
