# Supplementary material for: Single-polyp metabolomics reveals biochemical structuring of the coral holobiont at multiple scales
Source: Commun Biol. 2023 Sep 26;6:984. doi: 10.1038/s42003-023-05342-8 (PMC10522574; doi:10.1038/s42003-023-05342-8)
Supplement: Supplementary file 2 — Description of Supplementary Materials [file 42003_2023_5342_MOESM2_ESM.docx]

**Description of Additional Supplementary Files**

**File name:** Supplementary Data 1

**Description: Annotations of MS/MS spectra against the GNPS Library with molecular formula, molecular mass, mass error, precursor mass, ion source, charge, cosine scores and other relevant data.**

**File name:** Supplementary Data 2

**Description: Annotations of MS/MS spectra against the mzCloud Library with molecular formula, molecular mass, and match score.**

**File name:** Supplementary Data 3

**Description: Source Data For Figure 2C**
