# Supplementary material for: Single-polyp metabolomics reveals biochemical structuring of the coral holobiont at multiple scales
Source: Commun Biol. 2023 Sep 26;6:984. doi: 10.1038/s42003-023-05342-8 (PMC10522574; doi:10.1038/s42003-023-05342-8)
Supplement: Supplementary file 3 — Reporting Summary [file 42003_2023_5342_MOESM3_ESM.pdf]

nature portfolio

Corresponding author(s): Ty Roach

Last updated by author(s): 2023-5-9

Reporting Summary

Nature Portfolio wishes to improve the reproducibility of the work that we publish. This form provides structure for consistency and transparency in reporting. For further information on Nature Portfolio policies, see our Editorial Policies and the Editorial Policy Checklist.

Statistics

For all statistical analyses, confirm that the following items are present in the figure legend, table legend, main text, or Methods section.

n/a	Confirmed	
☐	☒	The exact sample size (*n*) for each experimental group/condition, given as a discrete number and unit of measurement
☐	☒	A statement on whether measurements were taken from distinct samples or whether the same sample was measured repeatedly
☐	☒	The statistical test(s) used AND whether they are one- or two-sided<br>*Only common tests should be described solely by name; describe more complex techniques in the Methods section.*
☐	☒	A description of all covariates tested
☐	☒	A description of any assumptions or corrections, such as tests of normality and adjustment for multiple comparisons
☐	☒	A full description of the statistical parameters including central tendency (e.g. means) or other basic estimates (e.g. regression coefficient) AND variation (e.g. standard deviation) or associated estimates of uncertainty (e.g. confidence intervals)
☐	☒	For null hypothesis testing, the test statistic (e.g. *F*, *t*, *r*) with confidence intervals, effect sizes, degrees of freedom and *P* value noted<br>*Give P values as exact values whenever suitable.*
☒	☐	For Bayesian analysis, information on the choice of priors and Markov chain Monte Carlo settings
☒	☐	For hierarchical and complex designs, identification of the appropriate level for tests and full reporting of outcomes
☐	☒	Estimates of effect sizes (e.g. Cohen's *d*, Pearson's *r*), indicating how they were calculated

Our web collection on statistics for biologists contains articles on many of the points above.

Software and code

Policy information about availability of computer code

Data collection

Randomized methanol extracts were analyzed on a ThermoTM QExactiveTM mass spectrometer coupled to a Vanquish Ultra High-Performance Liquid Chromatography (UHPLC) system. No other processing or purification of the extracted sample was performed except a centrifugation step for 30 seconds at 5000xg was used to pellet debris. A volume of 25 µl of the methanol extract containing the single polyp sample was added directly to a mass a 96-well sample plate and diluted 1:1 in 50% methanol containing an internal standard of 2.5 mg/ml phenol red. The mobile phase was 0.1% formic acid in Milli-Q water (channel A) and acetonitrile (channel B). The stationary phase was a reverse phase column Waters® Acquity® (Wood Dale, IL, USA) UPLC BEH C-18 column, 2.1 mm × 100 mm. The chromatographic runs were 12 min-long with linear gradients as follows: 0–1 min 2% B, 1–8 min 2–100% B. This 100% B solution was then held for 2 min followed by a switch to 2% B for the remaining 2 min. The injection volume was 10 µL, the flow rate 0.40 mL/min and the column temperature 60°C. Full MS1 survey scans and MS2 mass spectra for five precursor ions per survey scan were collected using electrospray ionization in positive mode with a scan range set from m/z 100 to 1500 for the full MS mode (minutes 1–10 of run). Quality control standards were also prepared from a random pool of 10 samples of the single polyp data. This QC mix was injected after every 12 samples of the MS run to monitor quality of the MS peaks and instrument performance. The mix was monitored throughout the run and a retention time drift from the first to last quality control standard peak was less than 0.01 minutes. Furthermore, an extraction blank containing our methanol extract but no coral sample was included at the time of sampling and through the entire MS procedure to monitor background signals in our reagent and instruments. These signals were removed from the resulting metabolome feature table based on their presence in blank samples compared to coral samples. A molecule had to be on average 3x higher in the sample than blank to be maintained in the data.
Raw files (.raw) were converted to .mzXML format for analysis. All files were processed with MZmine 2.53 software, the Global Natural Product Social web-based platform (GNPS) and SIRIUS (Dührkop et al. 2019; Pluskal et al. 2010; Wang et al. 2016). MZMine 2.53 parameters were set up as follows: feature extraction for MS1 and MS2 was performed for a centroid mass detector with a signal threshold of 5.0 x 104 counts. Chromatogram builder was run considering a minimum height of 1.0 x 105 and a m/z tolerance of 7 ppm. Chromatograms were deconvoluted with a peak duration range of 0.0 to 3.00 min and a baseline cut-off algorithm of 1.0 x 105. Isotopic peaks were grouped with a

m/z tolerance of 0.02 Da and a retention time percentage of 0.05. Detected peaks were aligned through Join Aligner Module considering 0.02 Da and retention time tolerance of 0.02 min. The resulting peak list was gap filled considering an intensity tolerance value of 0.001 ppm, 0.02 Da and retention time tolerance of 0.02 min. The data was converted to Mascot graphical format (.mgf) and a feature quantification table was generated for running feature-based molecular networking (FBMN) workflow on GNPS (Felix Nothias et al. 2019; Martin et al. 2019; Wang et al. 2016). This feature table included data from blank samples that were collected at the time of coral sampling using the same reagents and extraction solvents but did not contain a coral sample. Any metabolites detected in these blank samples had to be on average 3-times more abundant that those in blanks to be included in the feature table. After blank removal the abundance of each feature was normalized to the total feature abundances creating relative abundances for the metabolome data. FBMN was performed with a parent and fragment mass ion tolerance of 0.02 Da, a cosine score of 0.65 and a minimum matched peaks minimum of 4. Feature-based molecular networking job is available at: https://gnps.ucsd.edu/ProteoSAFe/status.jsp?task=3f5258d734374246a452591f23763b9f and raw files are available in MASSIVE (massive.ucsd.edu) as MSV000090806. The in silico molecular classification in Sirius was performed for Orbitrap instruments considering isotope scores and a mass deviation of 5 ppm. Molecular formulas were searched within biological databases only as well as the compound structural identification. CANOPUS was also applied for determining predicted compound class of relevant molecular features (cite https://pubmed.ncbi.nlm.nih.gov/33230292/). All molecules that were classified were compared across the dataset after summing the total abundances of each molecule at the Class level or most specific Class level according to the ClassyFire molecular classification scheme .

Data analysis

Discriminant analysis, principal component analysis, linear regressions, and comparison of means and standard deviations were performed in JMP14 or R statistical software. All analyses were run using default settings except discriminant analyses, which were run using JMP's preset "wide-linear, many column" setting. R package vegan was used to run PERMANOVAs (adonis2) and create PCOAs (Bray-Curtis distances). Linear mixed effects models were run using the R package lme4. Correlations coefficients between the CANOPUS ClassyFire molecular families and distance to base were calculated using Pearson's r value and p-values adjusted for multiple comparisons according to the Bonferonni method (see supplemental data files).

For manuscripts utilizing custom algorithms or software that are central to the research but not yet described in published literature, software must be made available to editors and reviewers. We strongly encourage code deposition in a community repository (e.g. GitHub). See the Nature Portfolio guidelines for submitting code & software for further information.

Data

Policy information about availability of data

All manuscripts must include a data availability statement. This statement should provide the following information, where applicable:

- Accession codes, unique identifiers, or web links for publicly available datasets
- A description of any restrictions on data availability
- For clinical datasets or third party data, please ensure that the statement adheres to our policy

All data for this project can be found at the following GNPS link: https://gnps.ucsd.edu/ProteoSAFe/status.jsp?task=3f5258d734374246a452591f23763b9f and the bleach mapping data is found at this GNPS link: https://gnps.ucsd.edu/ProteoSAFe/status.jsp?task=3f5258d734374246a452591f23763b9f
The data and EMBO ontology compatible metadata is publicly available at the GNPS MassIVE server at MassIVE ID: MSV000090806

Human research participants

Policy information about studies involving human research participants and Sex and Gender in Research.

Reporting on sex and gender	NA
Population characteristics	NA
Recruitment	NA
Ethics oversight	NA

Note that full information on the approval of the study protocol must also be provided in the manuscript.

Field-specific reporting

Please select the one below that is the best fit for your research. If you are not sure, read the appropriate sections before making your selection.

☐ Life sciences ☐ Behavioural & social sciences ☒ Ecological, evolutionary & environmental sciences

For a reference copy of the document with all sections, see nature.com/documents/nr-reporting-summary-flat.pdf

Ecological, evolutionary & environmental sciences study design

All studies must disclose on these points even when the disclosure is negative.

Study description

We present a method to detect high-quality metabolomic data from individual coral polyps and apply this method to study the patterning of biochemicals across multiple spatial (~1 mm - ~100 m) and organizational scales (polyp to population). For this study,

three branches were collected from each of 19 Montipora capitata colonies on a patch reef (21.451, --157.795) in Kāneʻohe Bay, Oʻahu, Hawaiʻi. Two branches were sampled from opposite sides of the colony, and one from the center (Figure 1A). Six single polyp biopsies were removed from each branch with a 16-gauge, blunt-tipped probe needle (Grainger) by sampling coral tissue directly surrounding an individual corallite to ensure the isolation of a single polyp. This resulted in a total dataset that contained 19 total colonies, 57 total branches (3 from each colony), and 342 polyps (6 from each brnach).

Research sample

Coral samples were collected in a single sampling event at patch reef #13 in Kāneʻohe Bay (21°30'49" N 157°55'03" W) on the windward side Oʻahu, Hawaiʻi. Three branches, approximately 8-15 cm in length, were collected from each of 19 Montipora capitata colonies, at the same depth (~3 m), via SCUBA diving. Branches were chosen based upon their location within M. capitata colonies: two from opposite extremities, and one from the center. Each colony was photographed before and after sampling. Additionally, each sampled branch was photographed, assigned a unique field ID, and had its location mapped within its colony of origin. Samples were then stored in seawater at ambient oceanic temperature and returned to the Hawaii Institute of Marine Biology (HIMB) for processing, where they were held in flow through seawater tanks (all single polyp samples were collected the same day as field collection in a single sampling event).

Sampling strategy

Six coral polyps were sampled from each coral branch for metabolomic analysis. Single polyp biopsies were removed by taking a tissue punch with a 16-gauge blunt tipped probe needle (Grainger) by sampling coral tissue directly surrounding the corallite to ensure the isolation of a polyp (Fig. 1 before/after tissue punch). The corallite was then removed from the needle by pushing air through a syringe directly into a 1.5 mL, amber glass vial containing 100 µl of 70% methanol solution. A blank sample was included using the same extraction solvent and sample vials but not coral sample. The samples were stored at -80°C. The following describes the polyp locations within a branch starting with "Polyp 1", 1 cm above the branch's base. The next two samples (Polyps 2 and 3) were taken from consecutive polyps following the first up the branch (Fig. 1G, three polyps sampled in a row). The first three samples were taken to assess the metabolites associated with three coral polyps directly next to one another in a colony. The next three samples were chosen based upon the distance between polyp 3 and the tip of the branch (Fig. 1G, distance of 3rd polyp to branch tip). Polyp 4 was sampled ¼ of the total distance from Polyp 3 to branch tip, and Polyp 5 was ½ of the total distance from Polyp 3 to branch tip. Lastly, Polyp 6 was taken from the very end of the branch tip (Fig. 1G polyps 4-6 labeled on branch). The last three samples were taken to assess the effect of isolation by distance on polyp metabolites within a single coral branch . All samples were taken in a randomized fashion to help eliminate bias and sampling artifacts; furthermore, samples were randomized prior to mass spectrometry analysis and given arbitrary labels to blind them from the analyst.

Data collection

All mass spec data was collected by the Quinn Lab using the following methodology. The randomized methanol extracts were analyzed on a ThermoTM QExactiveTM mass spectrometer coupled to a Vanquish Ultra High-Performance Liquid Chromatography (UHPLC) system. No other processing or purification of the extracted sample was performed except a centrifugation step for 30 seconds at 5000xg was used to pellet debris. A volume of 25 µl of the methanol extract containing the single polyp sample was added directly to a mass a 96-well sample plate and diluted 1:1 in 50% methanol containing an internal standard of 2.5 mg/ml phenol red. The mobile phase was 0.1% formic acid in Milli-Q water (channel A) and acetonitrile (channel B). The stationary phase was a reverse phase column Waters® Acquity® (Wood Dale, IL, USA) UPLC BEH C-18 column, 2.1 mm × 100 mm. The chromatographic runs were 12 min-long with linear gradients as follows: 0–1 min 2% B, 1–8 min 2–100% B. This 100% B solution was then held for 2 min followed by a switch to 2% B for the remaining 2 min. The injection volume was 10 µL, the flow rate 0.40 mL/min and the column temperature 60°C. Full MS1 survey scans and MS2 mass spectra for five precursor ions per survey scan were collected using electrospraelectrospray ionization in positive mode with a scan range set from m/z 100 to 1500 for the full MS mode (minutes 1–10 of run). Quality control standards were also prepared from a random pool of 10 samples of the single polyp data. This QC mix was injected after every 12 samples of the MS run to monitor quality of the MS peaks and instrument performance. The mix was monitored throughout the run and a retention time drift from the first to last quality control standard peak was less than 0.01 minutes. Furthermore, an extraction blank containing our methanol extract but no coral sample was included at the time of sampling and through the entire MS procedure to monitor background signals in our reagent and instruments. These signals were removed from the resulting metabolome feature table based on their presence in blank samples compared to coral samples. A molecule had to be on average 3x higher in the sample than blank to be maintained in the data.
Raw files (.raw) were converted to .mzXML format for analysis. All files were processed with MZmine 2.53 software, the Global Natural Product Social web-based platform (GNPS) and SIRIUS (Dührkop et al. 2019; Pluskal et al. 2010; Wang et al. 2016). MZMine 2.53 parameters were set up as follows: feature extraction for MS1 and MS2 was performed for a centroid mass detector with a signal threshold of 5.0 x 104 counts. Chromatogram builder was run considering a minimum height of 1.0 x 105 and a m/z tolerance of 7 ppm. Chromatograms were deconvoluted with a peak duration range of 0.0 to 3.00 min and a baseline cut-off algorithm of 1.0 x 105. Isotopic peaks were grouped with a m/z tolerance of 0.02 Da and a retention time percentage of 0.05. Detected peaks were aligned through Join Aligner Module considering 0.02 Da and retention time tolerance of 0.02 min. The resulting peak list was gap filled considering an intensity tolerance value of 0.001 ppm, 0.02 Da and retention time tolerance of 0.02 min. The data was converted to Mascot graphical format (.mgf) and a feature quantification table was generated for running feature-based molecular networking (FBMN) workflow on GNPS (Felix Nothias et al. 2019; Martin et al. 2019; Wang et al. 2016). This feature table included data from blank samples that were collected at the time of coral sampling using the same reagents and extraction solvents but did not contain a coral sample. Any metabolites detected in these blank samples had to be on average 3-times more abundant that those in blanks to be included in the feature table. After blank removal the abundance of each feature was normalized to the total feature abundances creating relative abundances for the metabolome data. FBMN was performed with a parent and fragment mass ion tolerance of 0.02 Da, a cosine score of 0.65 and a minimum matched peaks minimum of 4. Feature-based molecular networking job is available at: https://gnps.ucsd.edu/ProteoSAFe/status.jsp?task=3f5258d734374246a452591f23763b9f and raw files are available in MASSIVE (massive.ucsd.edu) as MSV000090806. The in silico molecular classification in Sirius was performed for Orbitrap instruments considering isotope scores and a mass deviation of 5 ppm. Molecular formulas were searched within biological databases only as well as the compound structural identification. CANOPUS was also applied for determining predicted compound class of relevant molecular features (cite https://pubmed.ncbi.nlm.nih.gov/33230292/). All molecules that were classified were compared across the dataset after summing the total abundances of each molecule at the Class level or most specific Class level according to the ClassyFire molecular classification scheme .

Timing and spatial scale

Coral samples were collected in a single sampling event at patch reef #13 in Kāneʻohe Bay (21°30'49" N 157°55'03" W) on the windward side Oʻahu, Hawaiʻi. Three branches, approximately 8-15 cm in length, were collected from each of 19 Montipora capitata colonies, at the same depth (~3 m), via SCUBA diving. Branches were chosen based upon their location within M. capitata colonies: two from opposite extremities, and one from the center. Each colony was photographed before and after sampling. Additionally,

each sampled branch was photographed, assigned a unique field ID, and had its location mapped within its colony of origin. Samples were then stored in seawater at ambient oceanic temperature and returned to the Hawaii Institute of Marine Biology (HIMB) for processing, where they were held in flow through seawater tanks (all single polyp samples were collected the same day as field collection in a single sampling event).
Six coral polyps were sampled from each coral branch for metabolomic analysis. Single polyp biopsies were removed by taking a tissue punch with a 16-gauge blunt tipped probe needle (Grainger) by sampling coral tissue directly surrounding the corallite to ensure the isolation of a polyp (Fig. 1 before/after tissue punch). The corallite was then removed from the needle by pushing air through a syringe directly into a 1.5 mL, amber glass vial containing 100 µl of 70% methanol solution. A blank sample was included using the same extraction solvent and sample vials but not coral sample. The samples were stored at -80°C. Samples were takenThe following describes the polyp locations within a branch starting with "Polyp 1", 1 cm above the branch's base. The next two samples (Polyps 2 and 3) were taken from consecutive polyps following the first up the branch (Fig. 1G, three polyps sampled in a row). The first three samples were taken to assess the metabolites associated with three coral polyps directly next to one another in a colony. The next three samples were chosen based upon the distance between polyp 3 and the tip of the branch (Fig. 1G, distance of 3rd polyp to branch tip). Polyp 4 was sampled ¼ of the total distance from Polyp 3 to branch tip, and Polyp 5 was ½ of the total distance from Polyp 3 to branch tip. Lastly, Polyp 6 was taken from the very end of the branch tip (Fig. 1G polyps 4-6 labeled on branch). The last three samples were taken to assess the effect of isolation by distance on polyp metabolites within a single coral branch . All samples were taken in a randomized fashion to help eliminate bias and sampling artifacts; furthermore, sSamples were randomized prior to mass spectrometry analysis and given arbitrary labels to blind them from the analyst.

Data exclusions	No data were excluded from this analysis
Reproducibility	Reproducibility was ensured by taking samples from all possible memebers of an in situ population and taking multiple branches per colony and mulitple polyps per branch.
Randomization	All samples were randomized prior to mass spectrometry analysis and given arbitrary labels to blind them from the analyst.
Blinding	All samples were randomized prior to mass spectrometry analysis and given arbitrary labels to blind them from the analyst.

Did the study involve field work? ☒ Yes ☐ No

Field work, collection and transport

Field conditions	All filed work was conducted on one sunny morning with light winds in Kaneohe Bay.
Location	patch reef 13 (21.451, --157.795) in Kāneʻohe Bay, Oʻahu, Hawaiʻi
Access & import/export	As we work at a field station in Hawaiithere was no import/ export needed. All work was conducted on an existing permit from HI DLNR (Permit #)
Disturbance	Three branches were clipped from each of 19 coral colonies in situ. To minimize any further disturbances these branches were taken back to the lab for the final single polyp samping.

Reporting for specific materials, systems and methods

We require information from authors about some types of materials, experimental systems and methods used in many studies. Here, indicate whether each material, system or method listed is relevant to your study. If you are not sure if a list item applies to your research, read the appropriate section before selecting a response.

Materials & experimental systems

n/a	Involved in the study	
☒	☐	Antibodies
☒	☐	Eukaryotic cell lines
☒	☐	Palaeontology and archaeology
☐	☒	Animals and other organisms
☒	☐	Clinical data
☒	☐	Dual use research of concern

Methods

n/a	Involved in the study	
☒	☐	ChIP-seq
☒	☐	Flow cytometry
☒	☐	MRI-based neuroimaging

Animals and other research organisms

Policy information about studies involving animals; ARRIVE guidelines recommended for reporting animal research, and Sex and Gender in Research

Laboratory animals	This study did not involve laboratory animals
Wild animals	This study involved 19 Montipora capitata coral colonies.
Reporting on sex	These organisms are hermaphroditic
Field-collected samples	Coral branches were then stored in seawater at ambient oceanic temperature and returned to the Hawaii Institute of Marine Biology (HIMB) for processing, where they were held in flow through seawater tanks (all single polyp samples were collected the same day as field collection in a single sampling event).
Ethics oversight	HI DLNR

Note that full information on the approval of the study protocol must also be provided in the manuscript.
